# Transmission Characteristics of 80 Gbit/s Nyquist-DWDM System in Atmospheric Turbulence

**DOI:** 10.3390/s25247598

**Published:** 2025-12-15

**Authors:** Silun Du, Qiaochu Yang, Tuo Chen, Tianshu Wang

**Affiliations:** 1 The National and Local Joint Engineering Research Center of Space Optoelectronics Technology, Changchun University of Science and Technology, Changchun 130022, China; silundu@mails.cust.edu.cn (S.D.); yangqc@cust.edu.cn (Q.Y.); 2 Department of Optical Engineering, School of Opto-Electronic Engineering, Changchun University of Science and Technology, Changchun 130022, China; chentuo@cust.edu.cn

**Keywords:** atmospheric turbulence, dense wavelength division multiplexing (DWDM), free-space optical (FSO) communication, nyquist pulse, transmission characteristics

## Abstract

We experimentally demonstrate an 80 Gbit/s Nyquist-dense wavelength division multiplexed (Nyquist-DWDM) transmission system operating in a simulated atmospheric turbulence channel. The system utilizes eight wavelength-tunable lasers with 100 GHz spacing, modulated by cascaded Mach–Zehnder modulators, to generate phase-locked Nyquist pulse sequences with a 10 GHz repetition rate and a temporal width of 66.7 ps. Each channel is synchronously modulated with a 10 Gbit/s pseudo-random bit sequence (PRBS) and transmitted through controlled weak turbulence conditions generated by a temperature-gradient convection chamber. Experimental measurements reveal that, as the turbulence intensity increases from $C_{n}^{2}=1.01\times 10^{-16}$ to $5.71\times 10^{-16}\;m^{-2/3}$, the signal-to-noise ratio (SNR) of the edge channel (C29) and central channel (C33) decreases by approximately 6.5 dB while maintaining stable Nyquist waveform profiles and inter-channel orthogonality. At a forward-error-correction (FEC) threshold of $3.8\times 10^{-3}$, the minimum receiver sensitivity is −17.66 dBm, corresponding to power penalties below 5 dB relative to the back-to-back condition. The consistent SNR difference (<2 dB) between adjacent channels confirms uniform power distribution and low inter-channel crosstalk under turbulence. These findings verify that Nyquist pulse shaping substantially mitigates phase distortion and scintillation effects, demonstrating the feasibility of high-capacity DWDM free-space optical (FSO) systems with enhanced spectral efficiency and turbulence resilience. The proposed configuration provides a scalable foundation for future multi-wavelength FSO links and hybrid fiber-wireless optical networks.

## 1. Introduction

The Nyquist laser carrier has been extensively utilized within optical networks to enhance spectral efficiency owing to its distinctive characteristics, such as a rectangular spectrum, as well as a periodic sinc-type pulse and concentrated energy in the time domain. It exhibits notable resilience against noise interference compared to Gaussian pulses [1,2,3]. Furthermore, the Nyquist pulse, with a narrow time-bandwidth product, enables extended transmission distances compared to other pulse types, reducing susceptibility to spatial link effects. Consequently, it demonstrates superior performance in the transmission of atmospheric channels. Building on these advantages, the integration of multiplexing techniques represents a natural evolution in optical communications. Over the past decade, the amalgamation of wavelength division multiplexing (WDM) and optical time-division multiplexing (OTDM) technologies has propelled the rapid development of high-capacity laser communication with a low bit error rate [4,5].

Following the early development of Nyquist pulse generation, a series of seminal works have demonstrated its potential for high-capacity optical-fiber transmission. In 2012, Hillerkuss et al. [6] digitally synthesized 325 Nyquist carriers and achieved 32.5 Tbit/s 16-QAM Nyquist-WDM transmission over 227 km of uncompensated single-mode fiber, confirming the spectral compactness and crosstalk suppression enabled by near-rectangular spectra. Around the same period, Nakazawa et al. [7] reported the first 160 Gbit/s Nyquist-OTDM system, showing that bandwidth utilization can be significantly enhanced compared with Gaussian pulses. Although Nyquist-OTDM benefits from a low peak-to-average-power ratio and strong nonlinear tolerance [8,9], the optical-domain shaping scheme used in these systems typically yields a roll-off factor of 0.5, limiting its spectral ideality. To overcome these constraints, Soto et al. [10] proposed a cascaded Mach–Zehnder Modulator (MZM) architecture for generating optical Nyquist pulses with practically zero roll-off, and later demonstrations using Kerr-frequency-comb sources enabled multi-channel Nyquist-WDM with excellent stability and sub-5% inter-channel intensity variation.

While these developments have solidified Nyquist pulse shaping as a powerful tool for fiber transmission, most existing work remains oriented toward guided-wave channels. Recent studies continue to improve phase stability, dispersion tolerance, and receiver DSP in fiber-based Nyquist-WDM systems. For example, through joint IQ-imbalance compensation and timing recovery [11], or dispersion-robust demultiplexing [12]. These efforts refine Nyquist transmission in optical fibers but do not address how Nyquist-shaped carriers behave in free-space channels subjected to turbulence-induced fading and phase distortions.

The DWDM has been introduced into FSO systems to extend fiber-link multiplexing techniques to wireless optics communication [13,14,15,16]. For instance, Huang et al. achieved 100 Tbit/s-level FSO transmission through three-dimensional multiplexing of wavelength, polarization, and OAM, laying an experimental foundation for multi-dimensional multiplexing systems [13]; Khalighi and Uysal, from a communication theory perspective, summarized the performance bottlenecks of FSO under turbulence fading, receiver noise, and link geometry constraints [14]; meanwhile, a 200 Gb/s WDM-FSO system based on directly modulated multi-wavelength TOSA demonstrated the feasibility of medium-distance high-speed transmission [15]; while Soumahoro et al. systematically studied the impact of different pulse waveforms on the performance of WDM links in FSO, providing an important reference for understanding the transmission behavior of traditional pulses under atmospheric turbulence conditions [16]. However, most existing WDM-FSO implementations still rely on Gaussian pulses, whose non-band-limited spectra contain long tails that are highly susceptible to turbulence-induced amplitude and phase fluctuations. Such distortions manifest as effective spectral leakage and inter-channel crosstalk in practical DWDM systems with finite adjacent-channel rejection. Singh et al. further reported that turbulence fading introduces pronounced SNR variations and BER degradation in Gaussian-WDM superchannels under different weather conditions, reinforcing the theoretical understanding that conventional pulse shapes are intrinsically more vulnerable to turbulence-enhanced spectral spreading [17]. In contrast, Nyquist pulses possess a strictly rectangular, band-limited spectrum that remains orthogonal when channels are spaced at the Nyquist interval, thereby offering inherent crosstalk suppression and improved spectral compactness. However, Singh et al.’s research was only at the stage of experimental simulation and did not conduct real transmission experiments to study the characteristics of Nyquist pulses in turbulent channels. Although this advantage becomes most evident in ultra-dense grids, commercially available DWDM components typically provide a minimum spacing of 100 GHz, which limits the observable performance difference in practical experiments. Other recent investigations on Nyquist-inspired modulation for FSO links [18,19,20,21] have mainly focused on theoretical modeling or DSP-based schemes rather than on experimental validation. 

Meanwhile, large-scale outdoor demonstrations by Matsuda et al. [22] have established record-capacity multi-aperture FSO systems, achieving up to 14 Tb/s over 220 m and setting important benchmarks for practical WDM-FSO performance. However, these field experiments primarily examine aggregate throughput in relatively stable outdoor conditions and do not probe the transmission dynamics of Nyquist-shaped carriers in different turbulence channels. Therefore, channel-resolved measurements of multi-channel Nyquist-DWDM transmission under controlled atmospheric turbulence are very limited in existing studies, which motivates the present experimental investigation. Thus, the present work experimentally characterizes Nyquist-DWDM propagation through tunable turbulence and evaluates whether Nyquist shaping can maintain channel uniformity and suppress turbulence-enhanced inter-channel interference in practical FSO systems.

This paper presents an experimental investigation into the transmission characteristics of a Nyquist-DWDM transmission system operating in turbulent channels. The system achieved a high transmission rate of up to 80 Gbit/s. The Nyquist pulse sequence, featuring a pulse width of 66.7 ps and a repetition frequency of 10 GHz, was multiplexed through DWDM using eight wavelength-tunable lasers modulated by MZMs. Following synchronous digital modulation, the Nyquist-DWDM pulse sequences were transmitted through three distinct weak turbulent channels. Notably, in Channels C29 and C33, the signal-to-noise ratio (SNR) of the demodulated eye diagrams was measured to be 16.78 dB, 13.34 dB, 10.49 dB, 15.54 dB, 12.92 dB, and 8.92 dB, respectively, under the conditions $C_{n}^{2}=1.01\times 10^{-16}\;m^{-2/3}$, $C_{n}^{2}=2.79\times 10^{-16}\;m^{-2/3}$, and $C_{n}^{2}=5.71\times 10^{-16}\;m^{-2/3}$. The system demonstrated a minimum sensitivity of −17.66 dBm in weakly turbulent channels.

## 2. Experimental Setup

**Figure 1:**
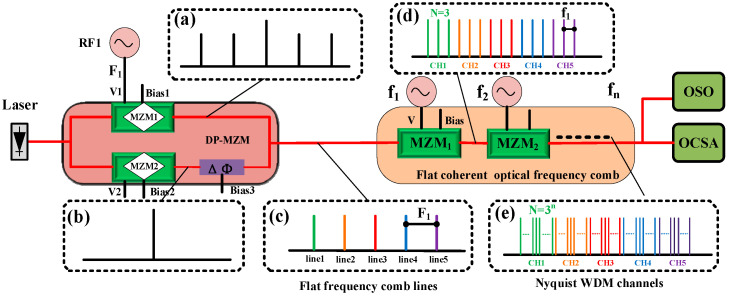
The generation mechanism of Nyquist pulses. (**a**) with modulated sidebands, (**b**) without sideband, (**c**) flat frequency comb, (**d**) pulse train with zero roll-off factor, (**e**) Nyquist-WDM channels.

As illustrated in Figure 1, the generation principle of the Nyquist pulse is based on a dual-parallel Mach–Zehnder modulator (DPMZM), which comprises two sub-modulators (MZM1 and MZM2) embedded in the upper and lower arms of a parent MZM. Two RF signals, together with two DC bias voltages, independently drive the two sub-MZMs, while an additional DC bias controls the optical-path-length difference between the two arms of the parent interferometer. In this configuration, a single RF signal (RF_1_) is applied to MZM1, whereas MZM2 is still not driven by any RF input. By appropriately adjusting V_1_ and Bias_1_, MZM1 produces first- and second-order optical sidebands with equal amplitudes, as shown in Figure 1a. Since MZM2 has no RF drive, only the optical carrier passes through it without introducing extra sidebands. When Bias_2_ and Bias_3_ are tuned properly, destructive interference occurs between the optical carriers from the two sub-MZMs, yielding a five-line flat optical frequency comb (OFC) as the DPMZM output. The frequency spacing between adjacent comb lines is equal to the modulation frequency of RF_1_ (f_1_), which can be continuously tuned. Each comb line of the DPMZM output can subsequently serve as an optical carrier for a WDM channel. By setting the modulation frequency to f_n_ = (1/3) f_n−1_ and cascading n MZMs, each carrier can generate an N = 3^n^ phase-locked and amplitude-flattened OFC, where N denotes the total number of comb lines and the frequency spacing equals f_n_, as illustrated in Figure 1d,e. According to the Fourier transform relationship, Equation (1) demonstrates that such a phase-locked OFC produces a sinc-shaped pulse train with a zero roll-off factor. When Δf = f_n_, the N-line phase-locked OFC corresponds to a Nyquist bandwidth of NΔf in the frequency domain and a sinc-shaped pulse train in the time domain with a repetition period of T = 1/Δf. The zero-crossing pulse width can thus be expressed as 2/(NΔf_n_). By setting the comb-line spacing of the DPMZM to F_1_ = NΔf, the desired Nyquist-WDM channels can be effectively synthesized.

(1) $$S\left(t\right)=\frac{sin(\pi N∆ft)}{N\;sin(\pi ∆ft)}$$

**Figure 2:**
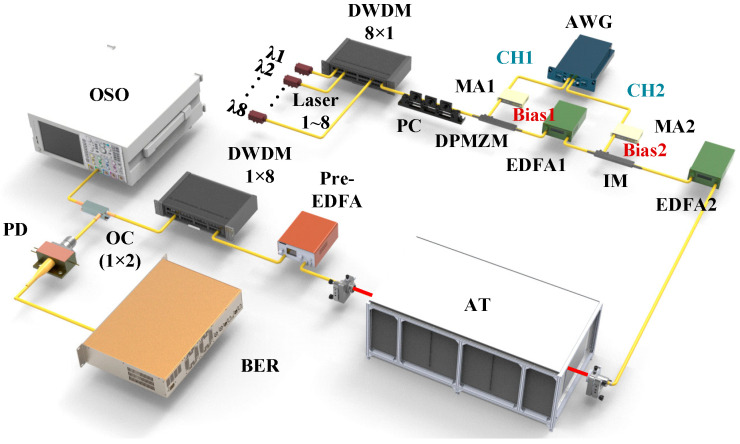
Schemes following the same experimental structure of 8-channel Nyquist-DWDM free-space optical communication.

The experimental structure of the Nyquist-DWDM turbulent channel transmission system is shown in Figure 2. Eight semiconductor lasers with a wavelength interval of 100 GHz were coupled to an 8 × 1 DWDM and then injected into a dual-parallel Mach–Zehnder modulator (DPMZM) for Nyquist pulse generation, as illustrated in Figure 2. The DPMZM was driven by the sinusoidal RF signal from Channel 1 of the AWG (Keysight Technologies, M8195A, Santa Rosa, CA, USA) to generate phase-locked Nyquist pulses with a repetition frequency of 10 GHz. The generated Nyquist-shaped multi-wavelength carriers were subsequently amplified by EDFA1 and modulated by a separate intensity modulator IM (FTM7939E) for data encoding using a pseudo-random bit sequence (PRBS) from Channel 2 of the AWG. It should be noted that the FTM7939E functions solely as the data modulation IM, while the DPMZM is responsible for Nyquist pulse generation. Channel 1 (CH1) of the arbitrary waveform generator transmitted a sinusoidal radio frequency (RF) signal, which was amplified by a microwave amplifier (MA1) and then drove the MZM. In addition, the amplitude of the RF signal was tuned, and the direct current (DC) bias voltage was set to $V=V_{\pi /2}$, which is the half-wave voltage of the modulator. A polarization controller (PC) was inserted before the modulator to control the polarization state of the laser. The obtained 8-channel Nyquist pulse was amplified using an Erbium-doped Fiber Amplifier 1 (EDFA) and implanted into a second intensity modulator to load a synchronous digital signal. The Nyquist-DWDM pulse sequence was modulated synchronously by PRBS generated by Channel 2 (CH2) of the AWG with the same modulation rate as the pulse repetition frequency. The modulated pulse signals were amplified by an EDFA2 and coupled into collimators on both sides of the turbulence channel simulator. A small-signal Erbium-doped fiber amplifier (Amonics, AEDFA-23-E-FA, Beijing, China) was applied to amplify the Nyquist-pulsed optical carrier after turbulent channel transmission. The carrier of each wavelength channel after DWDM demultiplexing was divided by a 50/50 optical coupler (OC), and the eye diagram and SNR of the Nyquist signal were observed using a high-speed optical sampling oscilloscope (Agilent, 86100C, Santa Clara, CA, USA). The system bit error rate (BER) was calculated by adopting off-line oscilloscope (Agilent, 86100C, Santa Clara, CA, USA) waveform data collected by the computer after avalanche photodiode detection.

**Figure 3:**
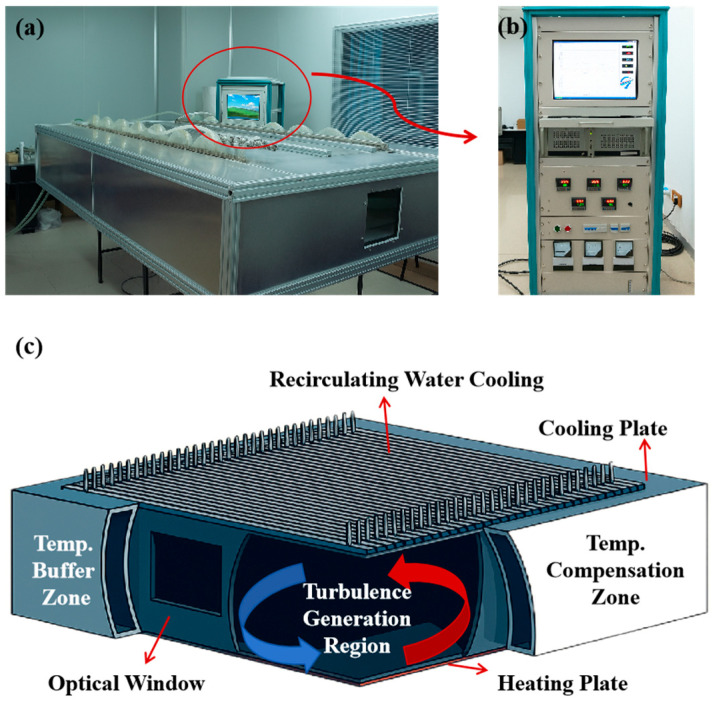
Simulated atmospheric turbulence device: (**a**) physical diagram, (**b**) simulated atmospheric turbulence controller, (**c**) structural decomposition of the atmospheric turbulence simulation pool.

Controllable turbulence intensity is essential in accurately characterizing the turbulence channel’s influence on the Nyquist-DWDM system’s transmission quality. An atmospheric turbulence simulator that can simulate weak turbulence channels was established based on the cold–hot air convection principle [23], as shown in Figure 3. The structure of the turbulence simulator consisted of a cooling plate, a heating plate, two optical windows, a temperature buffer zone, a turbulence zone, and a temperature compensation zone. The controller can feed back and control the temperature difference between the heating plate and the cooling plate.

The atmospheric coherence length ($r_{0}$) is a crucial parameter for quantifying turbulence intensity in atmospheric channels. It can be observed that the coherence length exhibits a decreasing trend with an increase in turbulence intensity [24,25]. In the experimental setup, a turbulent channel simulator was employed, and the atmospheric coherence length, following calibration, was expressed as follows:

(2) $$r_{0}=48\times {∆T}^{-0.81}$$

According to Equation (2), the atmospheric coherence length could be changed with the modification of temperature difference (∆T) between the heating and cooling plates, and thereby, the turbulence channel strength could be further controlled [26].

## 3. Experimental Results and Analysis

**Figure 4:**
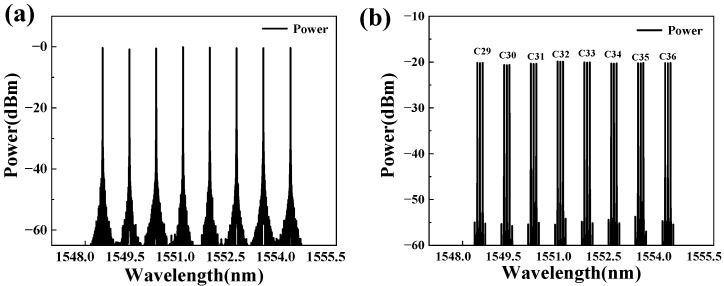
Simulated atmospheric turbulence device spectra of DWDM channels and Nyquist-DWDM channels: (**a**) spectrum of DWDM; (**b**) spectrum of MZM.

The output spectrum of the DWDM channel is shown in Figure 4a. The central wavelengths of eight semiconductor lasers (C29–C36) were 1548.52 nm, 1549.32 nm, 1550.12 nm, 1550.92 nm, 1551.72 nm, 1552.52 nm, 1553.32 nm, and 1554.13 nm, respectively. The adjacent channel interval was determined to be 0.8 nm, corresponding to a frequency of 100 GHz. The frequency of the sinusoidal signal generated by CH1 of AWG was determined to be 10 GHz, and the MZM was driven after amplification. In addition, the amplitude of the RF signal was adjusted to 0.6 V, and the DC bias voltage of the MZM was adjusted to 2.45 V, resulting in a phase-locked optical frequency comb for each channel. The Nyquist-DWDM spectrum of eight channels generated after MZM modulation is shown in Figure 4b. Each channel consisted of three phase-locked optical frequency combs with a rectangular spectrum distribution, and the comb tooth interval was equal to the modulation frequency f of sinusoidal RF signals. According to the correspondence between the optical frequency comb and Nyquist pulse [11], the zero-crossing pulse width was τ=2/N∆f (N is the number of teeth in the optical frequency comb). 

**Figure 5:**
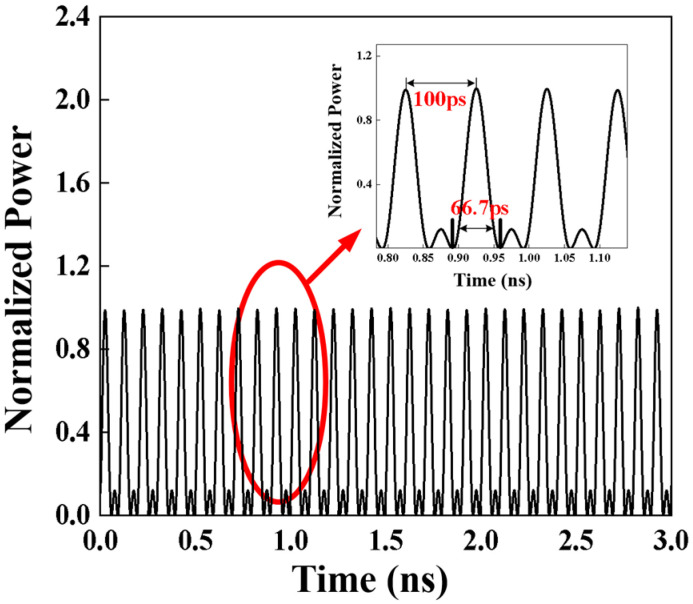
Nyquist pulse sequence generated by C29.

The time-domain pulse waveform of the C29 channel after 1 × 8 DWDM demultiplexing could be observed using a high-speed sampling oscilloscope, as shown in Figure 5. Three optical frequency combs with a frequency interval of 10 GHz could generate Nyquist pulses with a zero-crossing pulse width of 66.7 ps in the time domain.

The Nyquist pulse sequence generated exhibits a well-defined sinc-like temporal profile, confirming effective phase locking among the optical frequency comb lines. This temporal shape reflects the Fourier transform relationship between the rectangular spectral envelope and the time-domain sinc waveform, indicating that most of the optical energy is confined within the main lobe while the side lobes remain strongly suppressed. The narrow temporal width of 66.7 ps corresponds to a repetition frequency of 10 GHz, ensuring that the pulse sequence remains orthogonal between adjacent symbols, which is a key feature of inter-channel interference suppression in dense WDM systems.

Moreover, the stable and uniform Nyquist pulse train observed in the time domain suggests that the Mach–Zehnder modulator (MZM) bias and RF drive were accurately adjusted to achieve full constructive interference, minimizing amplitude fluctuation and phase jitter across channels. This high temporal coherence directly translates into reduced intersymbol interference (ISI) after digital modulation, thereby enhancing the signal integrity during transmission through turbulent channels. Consequently, the observed waveform verifies not only the fidelity of Nyquist pulse generation but also the robustness of the optical frequency comb-based shaping scheme for high-repetition-rate, high-spectral-efficiency optical communication.

**Figure 6:**
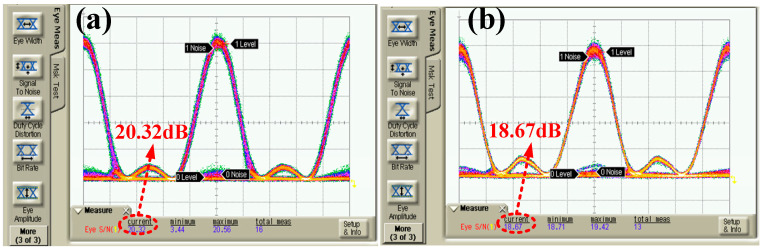
BtB Eye diagram: (**a**) Channel 29, (**b**) Channel 33.

**Figure 7:**
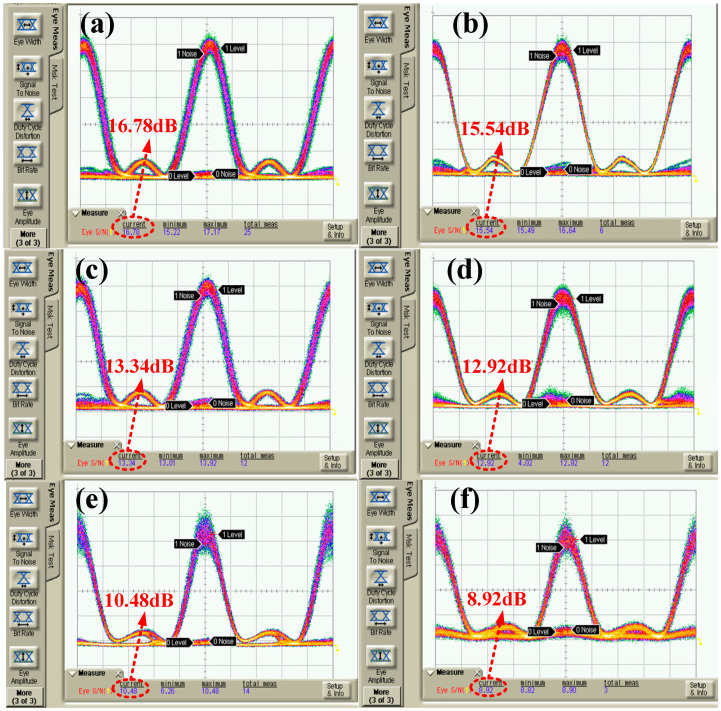
Eye diagrams: (**a**) 80 °C-C29; (**b**) 80 °C-C33; (**c**) 140 °C-C29; (**d**) 140 °C-C33; (**e**) 210 °C-C29; (**f**) 210 °C-C33.

The rate of the modulation channel should be synchronized with the repetition rate of the Nyquist pulse train, and the phase-matching condition should be met to ensure that the signal spectrum of each Nyquist-DWDM channel after digital modulation presents a rectangular shape. The PRBS bit rate of the CH2 channel output of the AWG was adjusted to 10 Gbit/s, and the modulation signal matched the pulse phase through the adjustable electrical delay line. The modulated Nyquist signal eye diagram was observed, applying a high-speed sampling oscilloscope with a bandwidth of 10 GHz, as shown in Figure 6. Under back-to-back (BtB) conditions, the Nyquist optical carrier signal-to-noise ratio of edge channel C29 and intermediate channel C33 had a maximum difference of 20.32 dB and 18.67 dB, respectively. The system had high noise tolerance and great modulation depth. In addition, the SNR difference between the two channels was 1.7 dB. The eye diagram and SNR of the Nyquist optical carrier in the C29 channel and C33 channel under three turbulence intensity conditions were measured after transmission in a turbulent channel with the obtained Nyquist-DWDM system, as shown in Figure 7. When the temperature difference between the heating plate and cooling plate of the simulated atmospheric turbulence device was ∆T=80 °C, ∆T=150 °C, and ∆T=210 °C, the corresponding turbulence intensity after calibration was $C_{n}^{2}=1.01\times 10^{-16}\;m^{-2/3}$, $C_{n}^{2}=2.79\times 10^{-16}m^{-2/3}$, and $C_{n}^{2}=5.71\times 10^{-16}\;m^{-2/3}$. The SNR of the C29 channel was 16.78 dB, 13.34 dB, and 10.48 dB under the three turbulence intensity conditions, and the SNR of the C33 channel was 15.54 dB, 12.92 dB, and 8.92 dB, respectively.

This behavior can be attributed to the combined influence of small-scale refractive index fluctuations and large-scale beam wander within the turbulent channel. The Nyquist pulse, characterized by a flat-top spectral distribution and precise temporal confinement, exhibits reduced sensitivity to phase-front distortion and amplitude scintillation compared with Gaussian-shaped carriers. The residual performance degradation mainly arises from localized intensity fading and the partial loss of spatial coherence at the receiver aperture. Because the DWDM channels are separated by 100 GHz, inter-channel crosstalk remains negligible, and the nearly constant SNR difference between C29 and C33 confirms that the multiplexed system preserves uniform power and phase balance under turbulence. These results demonstrate that the Nyquist-DWDM architecture maintains stable modulation depth and optical orthogonality across channels, ensuring reliable high-speed transmission through weakly turbulent free-space optical links.

As the channel turbulence intensity increases, the receiving optical eye diagram gradually deteriorates compared to the BtB condition, which also leads to a decrease in the SNR of the system by approximately 10 dB. The SNR difference between C29 and C33 channels did not exceed 2 dB at the same turbulence intensity. This difference resulted from the difference in the initial SNR of the two channels. With the increase in turbulence intensity, the “eyelid” gradually thickened; on the contrary, eye height was reduced, and therefore, the system noise tolerance decreased. However, when the turbulence intensity $C_{n}^{2}=5.71\times 10^{-16}\;m^{-2/3}$, the Nyquist waveform shape could still be maintained in the eye diagram of the C29 and C33 channels, which indicates the reliability of the Nyquist-DWDM system in a weak turbulence environment.

**Figure 8:**
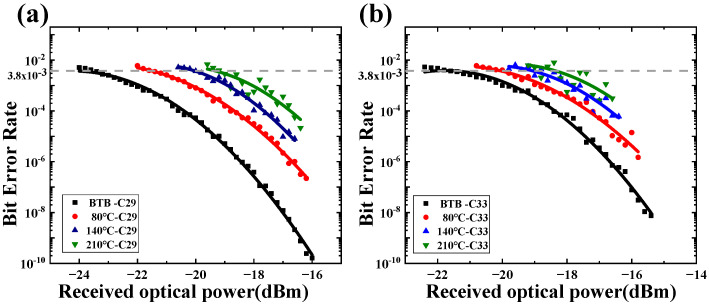
Bit error rate curves: (**a**) C29, (**b**) C33.

Figure 8 illustrates the BER of the Nyquist-DWDM system under different turbulence intensities. Figure 8a,b shows the BER curves of the C29 channel and C33 channel under the conditions of BtB $C_{n}^{2}=1.01\times 10^{-16}\;m^{-2/3}$, $C_{n}^{2}=2.79\times 10^{-16}\;m^{-2/3}$, and $C_{n}^{2}=5.71\times 10^{-16}\;m^{-2/3}$. When BER was the forward-error-correction limit $FEC=3.8\times 10^{-3}$, the minimum system sensitivities of the C29 channel and C33 channel under BtB conditions were −23.49 dBm and −21.07 dBm, respectively. The difference in receiving sensitivity between the two channels was 2.42 dB. When the turbulence intensities were $C_{n}^{2}=1.01\times 10^{-16}\;m^{-2/3}$, $C_{n}^{2}=2.79\times 10^{-16\;}m^{-2/3}$, and $C_{n}^{2}=5.71\times 10^{-16}\;m^{-2/3}$, the receiving sensitivities of the C29 channel were −21.01 dBm, −19.87 dBm, and −18.7 dBm, respectively. However, the receiving sensitivities of the C33 channel were −19.61 dBm, −18.12 dBm, and −17.66 dBm, respectively. The power penalties of the C29 and C33 channels were 4.79 dB and 3.41 dB, respectively, compared with that of BtB. The Nyquist-DWDM system with a channel interval of 100 GHz and single carrier rate of 10 Gbit/s was less affected by inter-channel crosstalk in a weakly turbulent channel, which was analyzed via BER curve comparison. 

The gradual degradation of receiver sensitivity and corresponding BER performance with increasing $C_{n}^{2}$ reflects the cumulative impact of turbulence-induced intensity fluctuations and phase distortion on the received optical field. As the turbulence strength increases, random variations in the refractive index cause scintillation and beam wander, which reduce the spatial coherence of the optical beam and lead to temporal power fading at the detector. However, the relatively moderate power penalty observed (<5 dB even at $C_{n}^{2}=5.71\times 10^{-16}{\;m}^{-2/3}$) indicates that Nyquist pulse shaping provides strong resilience against amplitude and phase noise. This resilience originates from the rectangular spectral profile of the Nyquist pulse, which maintains a uniform power distribution across frequency components and reduces intersymbol interference (ISI) under phase perturbations.

Furthermore, the lower level of degradation observed in the central channel (C33) compared with the edge channel (C29) suggests that the spectral edge channels experience slightly higher turbulence-induced spectral broadening and coupling loss, consistent with the greater effective divergence of off-axis wavelengths in free-space propagation. This asymmetry implies that channel equalization or adaptive power pre-compensation could further mitigate the observed performance gap.

Overall, the BER evolution across turbulence regimes confirms that the Nyquist-DWDM configuration enables stable transmission with minimal inter-channel crosstalk, demonstrating its suitability for weak-to-moderate turbulence environments. From a theoretical perspective, the reduced inter-channel crosstalk observed in Nyquist-DWDM systems originates from the strict orthogonality of the Nyquist pulse and its ideal rectangular, band-limited spectrum. Let p(t) denote the Nyquist pulse with a symbol period T. The transmitted signal of the k*-th* DWDM channel can be written as:

(3) $$s_{k}(t)=\sum_{n}a_{k,n}p(t-nT)e^{j2\pi f_{k}t}$$

where $f_{k}$ is the optical carrier frequency of channel k and $e^{j2\pi f_{k}t}$ is the optical carrier term of the k*-th* channel. After matched filtering and down-conversion at the receiver, the inter-channel interference term $C_{k\to i}\left(m-n\right)$ contributed by channel k to channel i is proportional to:

(4) $$C_{k\to i}\left(m-n\right)=\int p\left(\tau \right)\;p*\left(\tau -\left(m-n\right)T\right)e^{j2\pi \left(f_{k}-f_{i}\right)\tau }d\tau$$

The magnitude of this term depends on the spectral overlap between different channels. For a Nyquist pulse with an ideal rectangular spectrum P(f), where B is the bandwidth of Nyquist pulse can describe as follows [6,7,9]:

(5) $$P(f)=rect\;,(\frac{f}{B}),$$

The orthogonality condition is satisfied when the DWDM channel spacing meets the Nyquist criterion Δf=1/T. Under this condition, the spectral supports do not overlap:

(6) supp(P(f))∩supp(P(f−Δf))=∅,

Therefore,

(7) $$C_{k\to i}(m-n)\approx 0.$$

The channels are strictly orthogonal in the frequency domain. In contrast, Gaussian or raised-cosine pulses possess significant spectral tails and are not strictly band-limited; thus, spectral overlap persists even when the channel spacing equals 1/T, leading to non-zero crosstalk [15,16]. 

In FSO channels, atmospheric turbulence can be modeled as a stochastic transfer function H(f;ω) [23,24,25], which effectively “blurs” the spectrum of the transmitted signal. Prior studies have demonstrated that turbulence-induced spectral broadening severely degrades conventional WDM-FSO systems [14,25]. These degradations are primarily attributed to the enhanced spectral overlap of non-rectangular pulse shapes under turbulence.

In contrast, the finite-support rectangular spectrum of the Nyquist pulse ensures that the turbulence-distorted spectrum remains highly energy-concentrated, with limited spectral expansion. As a result, turbulence-enhanced inter-channel coupling is significantly suppressed. This theoretical behavior is consistent with our experimental observations: the inter-channel SNR difference in the proposed Nyquist-DWDM system remains below 2 dB under weak turbulence. These results confirm that Nyquist pulse shaping offers superior crosstalk resilience and maintains channel orthogonality even in turbulent atmospheric channels, highlighting the potential of Nyquist-DWDM techniques in free-space optical communication systems that require high spectral efficiency, channel stability, and robustness against atmospheric disturbances. 

It should be emphasized that, in the present implementation, the DWDM channel spacing is fixed at 100 GHz, which is determined by commercially available C-band multiplexers. Under such relatively loose spacing and weak turbulence conditions, the difference between Nyquist-shaped and conventional Gaussian-shaped pulses in terms of observable inter-channel crosstalk can be partially masked, because the channels are still well separated and the spectral overlap remains moderate. In other words, the full potential of Nyquist shaping for ultra-dense packing is not yet fully exploited. Table 1 calculates the spectrum leakage of adjacent channels for Gaussian pulses and Nyquist pulses under different channel spacings. As the channel spacing further decreases, the leakage of adjacent channels for Gaussian pulses shows an exponential growth trend. For a 10 Gb/s channel, the adjacent-channel leakage ratio of a Gaussian pulse increases to about −16 dB at a 10 GHz Nyquist spacing, whereas an ideal Nyquist pulse maintains zero out-of-band leakage at all spacings. This exponential sensitivity of Gaussian leakage to channel spacing highlights why Nyquist shaping becomes increasingly advantageous as the DWDM grid is made denser.

Therefore, from a theoretical standpoint, the rectangular and strictly band-limited spectrum of Nyquist pulses enables a higher spectral efficiency and inherently lower inter-channel interference than Gaussian pulses when the channel spacing approaches the Nyquist limit. In turbulence-impaired FSO links, this property is particularly attractive because any effective spectral leakage caused by turbulence-enhanced transfer-function fluctuations is enhanced when the signal spectrum is sharply confined. Therefore, even though the current experiment is constrained by the 100 GHz spacing of off-the-shelf DWDM modules, the demonstrated Nyquist-DWDM architecture provides a scalable platform: by migrating to finer grid spacings (e.g., 20 Ghz or below) in future work, the combined benefits of improved spectral efficiency and enhanced turbulence resilience can be more fully realized.

The scalability of the proposed Nyquist-DWDM architecture is grounded not only in its modular hardware design but more fundamentally in the optical-domain coherence of the DPMZM-generated Nyquist pulse train. Because Nyquist pulses are derived from a phase-locked optical frequency comb rather than purely digital shaping, their temporal and spectral orthogonality are preserved as channel count or symbol rate increases. The present experiment extends this framework by combining optical-domain Nyquist shaping with DWDM in a turbulent atmospheric channel, thereby showing that the inter-channel uniformity and Nyquist orthogonality persist under free-space propagation. Consequently, the setup is scalable to tens of channels or per-channel rates in the 25–40 Gbit/s class using standard C-band components, offering a credible path toward terabit-class FSO/hybrid fiber-wireless links while preserving uniform, low-crosstalk behavior.

## 4. Conclusions

In conclusion, an 80 Gbit/s Nyquist-DWDM transmission system operating under simulated atmospheric turbulence has been experimentally demonstrated, providing quantitative insights into the influence of turbulence on high-capacity, multi-channel optical links. The results show that although the SNR of both the edge channel (C29) and the central channel (C33) decreased by approximately 10 dB as the turbulence intensity increased, the Nyquist waveform remained well preserved even under the strongest weak turbulence condition. This observation confirms the inherent temporal and spectral robustness of Nyquist pulse shaping, which maintains channel orthogonality and effectively suppresses inter-channel crosstalk despite random refractive index fluctuations during propagation. Compared with conventional Gaussian-shaped WDM systems, the Nyquist-DWDM scheme offers distinct advantages in terms of spectral efficiency, channel orthogonality, and resilience to turbulence-induced phase distortion. Its rectangular spectral profile enables denser wavelength packing with minimal inter-channel crosstalk, while the sinc-shaped temporal waveform ensures stable signal transmission and enhanced robustness against amplitude and phase noise. These characteristics render the system particularly well suited for high-capacity optical wireless communication, where maintaining spectral compactness and uniform power distribution is critical.

Moreover, the measured minimum receiver sensitivity of −17.66 dBm at an FEC threshold of 3.8 × 10^−3^ verifies the feasibility of achieving stable, high-capacity transmission through weak turbulent channels. The small SNR variation (<2 dB) between adjacent channels indicates that power uniformity and phase stability can be effectively maintained in DWDM-based FSO systems when Nyquist pulse shaping is employed. These findings not only validate the compatibility of Nyquist-DWDM modulation with FSO communication architectures but also highlight its strong potential for scalable, multi-wavelength integration in future hybrid fiber-FSO networks. In addition, the demonstrated configuration shows promising potential for use in practical applications such as terabit-class FSO communication, satellite-to-ground and inter-satellite optical links, data center interconnects, and hybrid fiber-wireless systems that require both high capacity and environmental robustness. The future integration of Nyquist-DWDM modulation into frequency-comb sources, adaptive beam control, and intelligent equalization technologies may further extend its utility to cover 6G optical front-haul and broadband space-air-ground integrated networks. The demonstrated system provides a promising pathway toward terabit-class optical wireless communication with enhanced spectral efficiency, turbulence resilience, and overall link stability. Future research will focus on extending this approach to stronger turbulence regimes and integrating adaptive compensation techniques for dynamic beam control and real-time channel equalization, enabling more intelligent and robust high-speed FSO links.

## Figures and Tables

**Table 1:** Leakage of Adjacent Channels of Gaussian and Nyquist Pulse.

Channel Spacing Δ*f* (GHz)	Gaussian Leakage Ratio (η_Gaussian_)	Nyquist Leakage Ratio (η_Nyquist_)
10	2.38 × 10^−2^	0
12.5	1.41 × 10^−3^	0
15	3.32 × 10^−5^	0
20	1.03 × 10^−9^	0
50	≈1.0 × 10^−72^	0
100	≈3.0 × 10^−316^	0

## Data Availability

The original contributions presented in this study are included in the article. Further inquiries can be directed to the corresponding author.
